# Recurrence Quantification Analysis of F-Waves and the Evaluation of Neuropathies

**DOI:** 10.1155/2015/183608

**Published:** 2015-11-24

**Authors:** Morris A. Fisher, Vijaya K. Patil, Charles L. Webber

**Affiliations:** ^1^Department of Neurology, Hines VAH, Hines, IL 60141, USA; ^2^Department of Neurology, Loyola University Stritch School of Medicine, Maywood, IL 60153, USA; ^3^Department of Physiology, Loyola University Stritch School of Medicine, Maywood, IL 60153, USA

## Abstract

Electrodiagnostic (EDX) patterns of neuropathic dysfunction have been based on axonal/demyelinating criteria requiring prior assumptions. This has not produced classifications of desired sensitivity or specificity. Furthermore, standard nerve conduction studies have limited reproducibility. New methodologies in EDX seem important. Recurrent Quantification Analysis (RQA) is a nonlinear method for examining patterns of recurrence. RQA might provide a unique method for the EDX evaluation of neuropathies. RQA was used to analyze F-wave recordings from the abductor hallucis muscle in 61 patients with neuropathies. Twenty-nine of these patients had diabetes as the sole cause of their neuropathies. In the other 32 patients, the etiologies of the neuropathies were diverse. Commonly used EDX variables were also recorded. RQA data could separate the 29 patients with diabetic neuropathies from the other 32 patients (*P* < 0.009). Statistically significant differences in two EDX variables were also present: compound muscle action potential amplitudes (*P* < 0.007) and F-wave persistence (*P* < 0.001). RQA analysis of F-waves seemed able to distinguish diabetic neuropathies from the other neuropathies studied, and this separation was associated with specific physiological abnormalities. This study would therefore support the idea that RQA of F-waves can distinguish between types of neuropathic dysfunction based on EDX data alone without prior assumptions.

## 1. Introduction

The clinical classification of neuropathies has depended on electrodiagnostic (EDX) studies based on distinctions between axonal and demyelinating processes. Such an approach has limitations. Axonal and demyelinating injury is not dichotomous since axons and myelin are in fact intimately connected functionally. In addition, structural injury to nerves in a pathological sense is not the only basis for altered nerve conduction. Functional changes in ion channels can, for example, produce similar effects without disruption of the structural integrity of nerves.

As a methodology, axonal/demyelinating paradigms have been variable and have involved consensus criteria. Based on sensitivity and specificity, criteria sets using such paradigms have not produced a satisfactory EDX separation of acute (AIDP) and chronic (CIDP) inflammatory demyelinating polyneuropathies from other common neuropathies [1–3]. This is true despite almost 30 years of effort and at least 16 proposed criteria sets. This large number of criteria sets has been used to argue for the limited utility of the method in general [2]. A recent article reports that two of the proposed criteria sets are associated with a clinical diagnosis of CIDP with a reasonable degree of sensitivity and specificity [4]. This report, however, depends on prior clinical analysis and therefore retains the fundamental problem of all such studies. As has been argued elsewhere [3], it would be preferable to calculate the likelihood that a neuropathy has specific features based on the EDX data itself without dependence on a clinical diagnosis as the primary standard. These concerns about current approaches to EDX evaluation of neuropathies are compounded by the fact that adequate reproducibility of nerve conduction studies is present only if such studies are performed by the same electromyographer [5]. Given these issues, investigation of new techniques for EDX analysis is important and in theory could be rewarding given current computer capabilities.

F-waves are well established electrophysiological responses produced by antidromic activation (“backfiring”) of motoneurons [6]. F-waves are therefore affected by the normality or abnormality of the entire course of a motor nerve as well as by integrated central effects at the level of the motoneuron. They are characteristically analyzed following a series of supramaximal stimulations. They usually reflect discharge of one to several motor units and are therefore low in amplitude, usually less than 5% of the associated direct motor (M) response. F-waves are inherently variable in amplitude, latency, and configuration and may not appear after each stimulus. This variability with its potential richness of information as well as the long length of nerve monitored makes F-waves an attractive tool for defining patterns of nerve dysfunction.

This study describes the application of a nonlinear methodology (Recurrence Quantification Analysis [RQA]) [7] to the evaluation of F-waves. This nonlinear methodology allows one to quantitatively evaluate similarities versus differences in patterns of electrophysiological responses during a particular time during which that response is recorded. These data can then be sued to create a recurrence plot which is the graphical visualization of a square matrix in which the matrix elements correspond to those times at which a state of a dynamical system recurs. That is, the recurrence plot represents recurring patterns in time throughout the time of the signal evaluated. RQA methodology therefore provides a measure of complex changes during the period when the series of F-waves are recorded in what is an inherently dynamic and changing physiological environment. The results support the hypothesis that new, more automated modes of EDX analysis not dependent on prior assumptions could produce clinically meaningful information.

## 2. Methods

Tibial motor conduction studies and F-waves responses were recorded from the abductor hallucis (AH) muscle with techniques standard in the Clinical Neurophysiology Laboratory at the Hines VAH using the Laboratory EMG machines (Synergy, CareFusion, Natus, San Carlos, CA). The tibial nerve was chosen for analyses as one of the two most distal motor nerves accessible for EDX study. In contrast to the peroneal nerve, the tibial nerve is not affected by a common entrapment neuropathy (i.e., at the fibula head) nor as frequently affected by trauma in the region of the ankle.

All of the patients were recruited from an elderly veteran population and were male. The studies were performed by two of the authors (MF and VP). These studies were performed in 61 consecutive patients referred for evaluation of polyneuropathies to the Electrophysiology Laboratory at the Hines VAH and who were eligible for the study. All of the patients enrolled had length dependent sensorimotor polyneuropathies as determined by the recruiting physician. In addition, all of the patients had electrodiagnostic abnormalities in the tibial nerve studied that included at least abnormal slowing of conduction velocity as well as recordable F-waves (>20 *µ*V) from the AH. The diagnoses were based on the patients' histories, review of their medical records, and results of the entire electrodiagnostic examinations.

Twenty-nine of the patients had polyneuropathies in which the only etiological diagnosis was type II diabetes mellitus (*Group A*; mean age 66.0 years, range 50–87). Seven of the patients (*Group B*; mean age 66 years, range 58–82) had type II diabetes but also other diagnoses that could have contributed to their EDX abnormalities, namely, monoclonal gammopathy of uncertain significance (3), B12 deficiency (2), hepatitis C (1), and a lumbosacral radiculopathy (1). In the remaining 25 patients (*Group C*; mean age 57, range 53–84), the etiologies were diverse: alcoholism (4), B12 deficiency (4), monoclonal gammopathy of uncertain significance (4), chronic inflammatory demyelinating neuropathy (2), paraneoplastic (2), and hypothyroidism (2). In the remaining 7 of these 25 patients, the etiology for the neuropathies was not established. There were no statistically significant differences in the ages between the three groups. The limb, left or right, with the most prominent abnormalities in the tibial motor conduction studies was used for the analyses.

The F-waves were analyzed following 20 supramaximal stimuli, that is, 25% above that for eliciting a maximal direct motor (M) response. Stimuli were given at 0.5 Hz. F-wave variables analyzed included mean F latencies, F-wave persistence (i.e., the percentage of recordable F-waves), chronodispersion (i.e., the difference between the minimum and maximum F-wave latencies; CD), and mean minus predicted F-wave latencies (Fisher, 2003). Predicted mean F-latencies were determined using a regression equation including variables for height and age [8]. Mean latencies were used since they have consistently been shown to be preferable to minimal values (for references, see [9]). Other EDX variables were measured recording from the AH including distal motor latencies (DML); conduction velocities (CV); and compound motor action potential (CMAP) amplitudes and durations.

**Figure 1:**
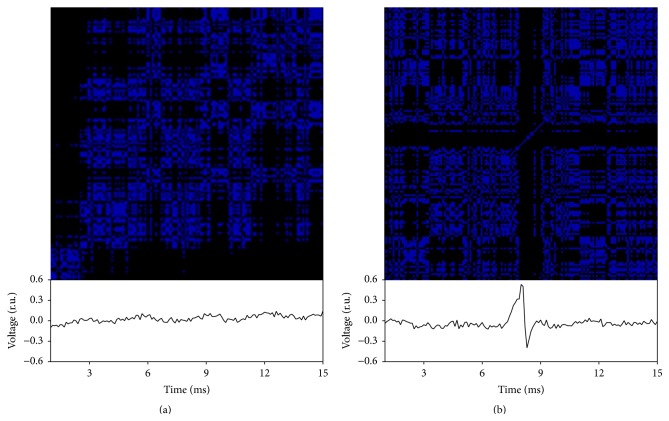
Recurrence plots of 15 ms time periods isolated from 150 ms recordings following nerve stimulation. F-waves were either absent (a) or present (b), but with different profiles when present. The recurrence plots show detailed distributions of recurrent points (blue areas) falling beneath an absolute radius threshold, namely, a predetermined threshold below which the point could be considered recurrent. Points making up the F-wave falling outside of the radius did not score recurrent points. The *x* and *y* axes represent time. As such for a particular point in time on the *x*-axis, a blue point on the *y*-axis (i.e., vertically above that time on the *x*-axis) would indicate recurrence points at subsequent times in the recordings.

**Figure 2:**
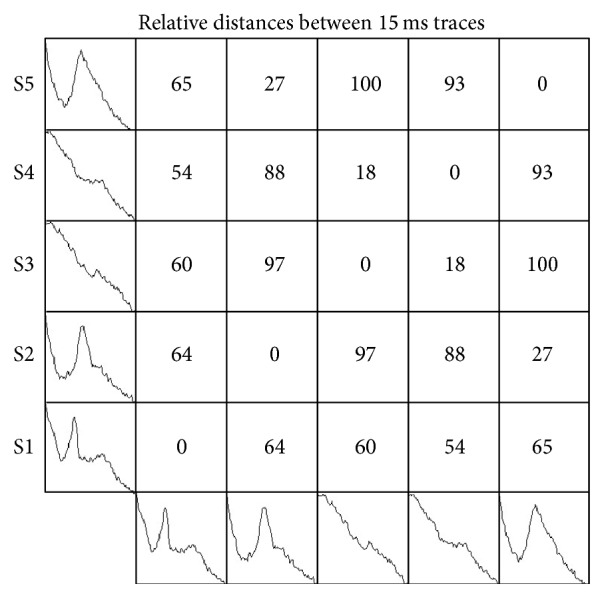
Schematic representation of the numeric designation of F-wave recurrence. Note that the numbers increase as the discrepancy between the configurations of the responses increases varying from 100 with marked differences to 0 for similar responses. These values were then used to determine which values were included in the recurrence matrix (the radius). As discussed in the text, lower values would be considered recurrent and higher values nonrecurrent. S1–5 represent individual F-wave recordings.

F-wave stimulation cycles were digitized at 10 KHz (Labscribe Data Acquisition System, IWorx, Durham, NH). Each subject was subjected to 20 stimuli delivered at 0.5 Hz and the nerve signal was recorded for 150 ms following each stimulus (0.1 ms resolution). F-wave contours were then isolated with shorter 15 ms windows as shown in Figure 1 for one subject. The 15 ms window was chosen so as to include that period of time when F-waves would be expected to occur. Some stimuli resulted in robust F-waves (Figure 1(a)) whereas other stimuli gave no reflex responses (Figure 1(b)). Representative recurrence plots for both are shown above the time series.  Figure 2 illustrates five stimulus cycles (S1, S2, S3, S4, and S5) showing how sequential F-wave profiles were assembled into a recurrence type of structure. The distances between paired profiles were computed as the sum of absolute point-for-point differences along the 150-point vectors which were then normalized over the unit interval (00–100%). F-waves with similar contours had low distances (e.g., 18% for S3-S4) whereas dissimilar F-waves had high distances (e.g., 100% for S1–S3). Zero distances were always scored along the diagonal where F-waves were compared with themselves (redundant). For this study, the F-wave recurrence plots consisted of 20 stimulus cycles or 400 pairings. Low distances were rendered as highly recurrent (similar contours) whereas high distances were rendered as lowly recurrent (dissimilar). The RQA radius cutoff was set such that the recurrence density was low (sparse matrix below 20% recurrent points) assuring that only the most similar F-wave pairing scored as being recurrent.

The upper triangle of recurrent points, excluding the ubiquitous line of identity, was subjected to Recurrence Quantification Analysis. Following the definitions of Webber Jr. and Zbilut [7], the patterning of recurrences was captured by 8 recurrence variables: REC (density of recurrence points in the plot); DET (determinism or proportion of recurrent points forming diagonal lines); DMAX (longest diagonal line); ENT (line-length entropy or signal complexity); TREND (measure of signal stationarity); LAM (laminarity or proportion of recurrent points forming vertical lines); TT (trap time or the mean length of vertical lines); VMAX (longest vertical line). These 8 variables have been demonstrated to reveal hidden, nonlinear characteristics in complex dynamics not captured by standard linear techniques such as time series or spectral methodologies.

Statistical analyses were performed using Student's *t*-tests, Fisher's exact test, and Mann-Whitney tests. Possibly statistically significant differences were noted for *P* < 0.05. Strong trends were noted for *P* = 0.05 to <0.10. Given the methodology and repeated analyses of the same data, statistically significant differences would be considered present with a Bonferroni correction if *P* < 0.02.

The study was approved by the IRB Committee at the Edward Hines Jr. VA Hospital.

## 3. Results

There were no statistically significant differences for the eight individual RQA variables between each of the three groups (A, B, and C). All of the *P* values between the three groups were greater than 0.10. %REC/%LAM ratios >0.35, however, were present in 19 of the 36 patients (53%) with diabetes (Groups A and B) in comparison to 6 of the 25 patients (24%) without diabetes in Group C (*P* < 0.035). In Group A alone (i.e., patients with diabetes as the only etiology for the neuropathy), 17 of the 29 patients (59%) had %REC/%LAM ratios >0.35. This was significantly different than the ratio for Group C patients without diabetes (*P* < 0.014). In the 7 patients in Group B (i.e., patients with diabetes as well as other possible causes for a neuropathy), only 2 had %REC/%LAM ratios values >0.35. The difference in the %REC/%LAM ratios >0.35 between Group A patients and all of the other neuropathies (Groups B and C) was *P* < 0.009. Based on %REC/%LAM values, there was no meaningful difference between Group B and the other two groups, but the number in Group B was small (*n* = 7). The *P* values for differences in %REC/%LAM values for the three groups are shown in Table 1. No statistically significant differences were present for any ratios of the other six RQA variables analyzed other than for %REC/%LAM discussed above.

Table 1 also shows the EDX variables with *P* values <0.10 (i.e., statistically significant or strong trends) when recording from the AH following stimulation of the tibial nerve. The data for these EDX variables are shown in Table 1. Other than for CMAP amplitudes and F-wave persistence, no statistically relevant *P* values were found; that is, all *P* values comparing the groups were >0.10. As can be seen in Table 1, CMAP amplitudes were lower at a statistically significant level in Group A in comparison to the other two groups. This was also true for F-wave persistence. Although the mean conduction velocities were close to the lower limit of normal (40 m/sec), 75% of these values were abnormal and the remainder were close to the lower limit of normal; the relatively large standard deviations were due to some values that were prominently slowed, for example, 21.5 m/sec. For all of the variables with statistically significant differences shown in Table 1 (CMAP amplitudes, F-wave persistence, and %REC/%LAM > 0.035), these differences were most prominent when the data from Group A (i.e., patients with only diabetes mellitus as a cause for their neuropathy) were compared with all of the other 32 patients (all *P* values <0.009).

## 4. Discussion

These results would support the idea that RQA analysis of F-wave studies can distinguish between patients with diabetes and neuropathies in comparison to studies from patients with other etiologies for their neuropathies. The patients were sorted based on the etiology of their neuropathies. Given the patient population with its high percentage of patients with diabetes mellitus, the only reasonable experimental approach in this study was to see if the RQA methodology could separate those with diabetic neuropathies from those with neuropathies of other causes. Although the number of patients with diabetes and other possible causes for their neuropathies (Group B) was small (*n* = 7), the data would be encouraging for thinking that the method could separate such patients from those patients with diabetes as the sole cause for their neuropathies (Group A). Indeed, the most statistically significant data were present when the data from Group A (i.e., patients with diabetes as the sole diagnosis) was compared with data from all the other patients including those in Group B (i.e., patients with diabetes but also other possible etiologies for their neuropathies).

The patients with only diabetes as a cause for their neuropathies had lower CMAP amplitudes. Combined with decreased F-wave persistence [10, 11], it could be argued that the separation of the groups evaluated was therefore due to a more “axonal” pattern of dysfunction in Group A patients. Although this may reflect a sampling factor due to the patients studied, there is little reason to think based on past experience that in general an “axonal” pattern of neuropathic injury* per se* could separate diabetic neuropathies from a comparable number of neuropathies randomly referred to an EDX laboratory. The critical issue here, however, is that RQA analysis of F-waves appeared able in a statistically significant manner to separate diabetic neuropathies from other types of neuropathies. Given this, the CMAP amplitude and F persistence data in this study would support the validity of the RQA methodology in that the RQA findings were then associated with specific, definable pathophysiological dysfunction. Indeed since the fundamental analysis involved evaluation of F-waves and F-wave variables are related to other EDX variables, it would be surprising if there was not some correlation with more traditional EDX variables. A lack of such a correlation could even raise questions about the validity of the method. The relationship between the RQA findings and traditional EDX variables do not diminish the value of the finding that diabetes could produce a particular type of neurogenic dysfunction characterized in part by a pattern of increased concentration of F-wave recurrences within a specified radius (i.e., increased %REC) and/or a decreased linear array (decreased %LAM) of such recurrences (i.e., decreased similar recurrences at the same point in time of each 15 ms of F-wave recordings). This could be consistent with neurogenic dysfunction in diabetes producing a relatively specific pattern of EDX findings as has been reported previously [12]. The potential is that further studies would show that other types of neuropathies would be associated with other patterns of RQA findings. In addition, the RQA results could provide guidance as to which standard EDX variables should be emphasized when evaluating different types of neuropathies.

The RQA methodology is based on evaluation of F-waves. It may not be surprising if analysis of F-waves provided a sensitive basis for classifying nerve dysfunction. F-waves are characteristically abnormal in neuropathies and may be more sensitive than conventional motor conduction studies [13]. F-wave latencies are the most stable and reliable measurements to evaluate sequential EDX examinations in the same subject [14]. This may be true because of the extended length of a particular nerve evaluated, that is, from the distal site of stimulation to the spinal cord. Unlike most other commonly used studies in EDX examinations, F-waves have a more variable complexity and therefore the potential for a greater degree of information. In addition, the RQA methodology measures changes over time in what is in fact a constantly changing physiological substrate. This is particularly relevant for F-waves since these responses are dependent on the constantly changing physiology affecting the anterior horn cells in the spinal cord.

Although the number of patients in this study was not small, further evaluation of the method would require considerably more patients with differing types of neuropathies and careful control data. This would probably ultimately require input from a number of EDX laboratories. This will not occur unless there is recognition of the importance of such work and some evidence to indicate such efforts could be rewarding. More than arguing for any specific new methodology, it is in that context that this work is presented. In that context, it is also worth emphasizing that the ability of the RQA methodology to separate neuropathies in patients with diabetes was serendipitous. The ability of the %REC/%LAM values to separate on the basis of this ratio being greater or less than 0.35 resulted from analysis of the data and not from any idea as to what should be present or initial hypothesis. The technique in theory could be useful both for defining the type of neuropathy in an individual patient and for comparing groups.

Whatever the limitations of this study, the data would support a proof of hypothesis. The data supports the potential value of new methods for analyzing EDX data and could indicate that RQA could be a powerful tool for this. This study is not meant to imply, however, that RQA is necessarily the only such tool or even necessarily the best tool. What is important is that this study could encourage the idea that meaningful clinical distinctions can be made based on EDX data alone without the need to fit such data into* a priori* criteria. Such methods could be particularly potent if data from more than one nerve were analyzed and potentially even using different methods for evaluating the same data. Despite the ready availability of increasingly powerful computers, there have been few such studies analyzing nerve conduction data based on such a concept. Such studies would seem particularly important in a field that arguably has not maintained its technological edge and where traditional patterns of thinking have not necessarily fulfilled their initial promise.

## Figures and Tables

**(a):** 

	Group A	Group B	Group C
Number	29	7	25
Distal motor latency	4.8 ± 0.86 ms	5.0 ± 0.6	4.7 ± 1.0
Conduction velocity	37 ± 5.4 m/sec	40.4 ± 2.6	37.5 ± 4.4
CMAP amplitude	3.9 ± 3.9 mV	8.4 ± 5.1	6.0 ± 3.9
CMAP duration	9.0 ± 4.8 ms	6.5 ± 2.7	8.2 ± 3.2
Mean F-latency	69.4 ± 6.0 ms	64.2 ± 7.3	67.1 ± 9.2
Mean/predicted F-latency	1.3 ± 0.3	1.2 ± 0.1	1.2 ± 0.1
F-wave chronodispersion	8.7 ± 3.6 ms	8.0 ± 3.7	9.5 ± 3.8
F-wave persistence (%)	75.8 ± 24.6	99.0 ± 2.6	90.2 ± 13.2
%REC/%LAM	0.38 ± 0.20	0.35 ± 0.28	0.32 ± 0.31

Values mean ± standard deviation.

**(b):** 

Groups	A versus C	B versus C	A versus B	A and B versus C	A versus B and C
CMAP amplitudes^*∗*^	*P* < 0.016	NS (*P* = 0.24)	*P* < 0.013	*P* < 0.067	*P* < 0.007
F-wave persistence^*∗*^	*P* < 0.043	*P* < 0.010	*P* < 0.020	*P* < 0.093	*P* < 0.001
%REC/%LAM >0.35^*∗∗*^	*P* < 0.014	NS (*P* = 0.60)	NS (*P* = 0.20)	*P* < 0.035	*P* < 0.009

Group A: diabetic neuropathies; Group B: diabetes and other diagnoses; Group C: nondiabetic neuropathies.

^*∗*^ *t*-tests.

^*∗∗*^Mann-Whitney test.

## Abbreviations

AH: Abductor halluces
AIDP: Acute inflammatory demyelinating polyneuropathy
CD: Chronodispersion
CIDP: Chronic inflammatory demyelinating polyneuropathy
EDX: Electrodiagnostic
LAM: Laminarity
REC: Recurrence
RQA: Recurrence Quantification Analysis.
